# Repair Effect of Seaweed Polysaccharides with Different Contents of Sulfate Group and Molecular Weights on Damaged HK-2 Cells

**DOI:** 10.3390/polym8050188

**Published:** 2016-05-19

**Authors:** Poonam Bhadja, Cai-Yan Tan, Jian-Ming Ouyang, Kai Yu

**Affiliations:** 1 Department of Chemistry, Jinan University, Guangzhou 510632, China; poonambhadja@gmail.com (P.B.); cy919889326@163.com (C.-Y.T.); yiyanget@163.com (K.Y.); 2 Institute of Biomineralization and Lithiasis Research, Jinan University, Guangzhou 510632, China

**Keywords:** seaweed, cell repair activity, polysaccharide degradation, molecular weight, cytotoxicity

## Abstract

The structure–activity relationships and repair mechanism of six low-molecular-weight seaweed polysaccharides (SPSs) on oxalate-induced damaged human kidney proximal tubular epithelial cells (HK-2) were investigated. These SPSs included *Laminaria japonica* polysaccharide, degraded *Porphyra yezoensis* polysaccharide, degraded *Gracilaria lemaneiformis* polysaccharide, degraded *Sargassum fusiforme* polysaccharide, *Eucheuma gelatinae* polysaccharide, and degraded *Undaria pinnatifida* polysaccharide. These SPSs have a narrow difference of molecular weight (from 1968 to 4020 Da) after degradation by controlling H_2_O_2_ concentration. The sulfate group (–SO_3_H) content of the six SPSs was 21.7%, 17.9%, 13.3%, 8.2%, 7.0%, and 5.5%, respectively, and the –COOH contents varied between 1.0% to 1.7%. After degradation, no significant difference was observed in the contents of characteristic –SO_3_H and –COOH groups of polysaccharides. The repair effect of polysaccharides was determined using cell-viability test by CCK-8 assay and cell-morphology test by hematoxylin-eosin staining. The results revealed that these SPSs within 0.1–100 μg/mL did not express cytotoxicity in HK-2 cells, and each polysaccharide had a repair effect on oxalate-induced damaged HK-2 cells. Simultaneously, the content of polysaccharide –SO_3_H was positively correlated with repair ability. Furthermore, the low-molecular-weight degraded polysaccharides showed better repair activity on damaged HK-2 cells than their undegraded counterpart. Our results can provide reference for inhibiting the formation of kidney stones and for developing original anti-stone polysaccharide drugs.

## 1. Introduction

Kidney stone is a common disease which occurrence is closely related to the damage of renal tubular epithelial cells. A high concentration of oxalate in the urine can damage renal tubular epithelial cells to trigger a series of oxidative stress reactions and eventually lead to the formation of kidney stones [1,2]. Urinary glycosaminoglycans (GAGs) can protect renal tubular epithelial cells against oxidative damage [3,4] and thus inhibit the formation of calcium oxalate stones.

Seaweed polysaccharides (SPSs) comprise repeating disaccharide sugar chains, similar to GAGs. Therefore, SPSs may be used to repair damaged renal tubular epithelial cells and inhibit the formation of stones. Polysaccharides from the seaweeds *Fucus* [5] and *Sargassum* [6] reportedly play significant protective roles in renal-tissue injury.

Faggio *et al.* [7] evaluated the anticoagulant activity of two algal sulfate polysaccharides extracted from *Ulva fasciata* (Chlorophyta) and *Agardhiella subulata* (Rhodophyta). No cytotoxicity was observed for the two SPS on human red blood cells, and both of them had significant anticoagulant activity. Polysaccharide extracted from brown alga *Undaria pinnatifida* also has an anticoagulant effect on human blood *in vitro* and are not cytotoxic [8].

The biochemical activity of SPS is determined by its chemical structure, especially molecular weight, as well as the content of sulfate groups and uronic acid [9,10,11,12]. In general, low-molecular-weight SPSs show effective biological activities. Zhao *et al.* [10] confirmed that low-molecular-weight degraded *Porphyran* polysaccharide has stronger scavenging ability of hydroxyl radical and DPPH radical.

Additionally, the higher content of anionic groups of polysaccharide such as the sulfate and uronic acid group is related to the beneficial biological activity. The *Laminaria* polysaccharide with higher uronic acid displayed stronger antioxidant activity [10]. Zhang *et al.* [13] and Wang *et al.* [14] also found that the high sulfate content of polysaccharide significantly increased its antioxidant activity. The –SO_3_H content of polysaccharides extracted from *Ascophyllum nodosum* at 150, 120, and 90 °C for 30 min were 7.82%, 16.87%, and 28.60%, respectively [15], and their scavenging capacity to DPPH radical is proportional to their –SO_3_H content, *i.e.*, the component with 28.60% –SO_3_H content has the highest scavenging capacity of about 15%, whereas the component with 7.82% –SO_3_H content only has scavenging capacity of about 2%. Wang *et al.* [12] reported that sulfated polysaccharides from *Cyclocarya paliurus* present significantly higher repair capacity on H_2_O_2_-induced damaged RAW264.7 cells than non-sulfate polysaccharide samples. To date, few reports exist about the repair effect of SPSs on oxalate-induced cytotoxicity, especially the structure–activity relationships remain unknown.

To comparatively investigate the structure–activity relationships of SPSs and their repair ability on injured renal epithelial cells, in the present work, we selected six low-molecular-weight SPSs with narrow differences but various acidic sulfate group (−OSO_3_^−^) content (21.7%, 17.9%, 13.3%, 8.2%, 7.0%, and 5.5%, respectively). We focused on studying the effect of –SO_3_H content of SPSs on their ability to repair injured human kidney proximal tubular epithelial cells (HK-2). Our results can provide reference for inhibiting the formation of kidney stones and for developing original anti-stone polysaccharide drugs.

## 2. Experimental Methods

### 2.1. Reagents and Apparatus

*Laminaria japonica* polysaccharide (*Laminaria*-1), *Porphyra yezoensis* polysaccharide (*Porphyra*-2), *Gracilaria lemaneiformis* polysaccharide (*Gracilaria-3*), *Sargassum fusiforme* polysaccharide (*Sargassum-4*), *Eucheuma* polysaccharide (*Eucheuma-5*), and *Undaria pinnatifida* polysaccharide (*Undaria-6*) were produced by Beijing newprobe Bioscience & Technology Co., Ltd (Beijing, China).

The cell proliferation assay kit (Cell Couting Kit 8, CCK-8) was purchased from Dojindo Laboratory (Kumamoto, Japan). Other chemical reagents were analytical grade and purchased from Guangzhou chemical reagent company (Guangzhou, China).

The apparatus included a Ubbelohde capillary viscometer (0.4–0.5, Qihang Glass Instrument Factory, Shanghai, China), an Ultraviolet-Visible spectrophotometer (Cary 500, Varian, Palo Alto, CA, USA), a Fourier Transform Infrared Spectrometer (EQUINOX 55, Bruker, Karlsruhe, Germany), a conductivity meter (DDS-11A, LEICI, Shanghai, China), Enzyme Mark Instrument (Safire2, Tecan, Männedorf, Switzerland), and an upright electric fluorescence microscope (22DI-E-D282, Leica, Solms, Germany).

### 2.2. Degradation of Polysaccharides

According to the molecular weight of natural polysaccharides, H_2_O_2_ was used to degrade high-molecular-weight SPSs. Taking *Porphyra*-2 for example, the degradation process was as follows. About 1.2 g of *Porphyra*-2 was weighed accurately and dissolved in 40 mL of distilled water at 70 °C. After heating to 90 °C, 27 mL of 30% (vol) H_2_O_2_ solution was quickly added to the reaction system. The degradation reaction was allowed to proceed for 2 h, at which point the solution pH was adjusted to 7.0 by adding 2 mol/L NaOH solution. The degraded SPS solution was then concentrated to one-third of its original volume at 60 °C. The product was precipitated by three times addition of anhydrous ethanol to the actual volume. The solution was stored at 4 °C in a refrigerator overnight and then filtered. The filtrate was washed with anhydrous ethanol twice and dried in a vacuum.

The degradation conditions of all the six SPSs are listed in Table 1.

### 2.3. Average Molecular Weight Determination

According to reference [16], the Ubbelohde viscosity method was used to measure the viscosity of samples in aqueous solutions at 30 ± 0.2 °C. After measuring the falling time in the viscometer before and after polysaccharide degradation, relative (η_r_) and specific (η_sp_) viscosity could be calculated from η_r_ = *T*_i_/*T*_0_ and η_sp_ = η_r_ − 1, where *T*_i_ and *T*_0_ are the falling time of SPS solutions and deionized water, respectively. Intrinsic viscosity (η) could be calculated according to the one-point method, *i.e.*, η = (2(η_sp_ − lnη_r_))^1/2^/*c*, in which *c* is the concentration of samples to be tested. The molecular weight of SPS was calculated through its value η. The relationship between η and the molecular weight *M* of the polymer solutions could be described by the Mark–Houwink empirical equation η = κ*M*^α^, where κ and α are constants related to polymer nature, solvent, and temperature.

### 2.4. Measurement of Protein Content

#### 2.4.1. Ultraviolet-Absorption Method

Polysaccharide solutions were scanned at a UV–VIS spectrophotometer to observe absorption situation between 260 and 280 nm. If the polysaccharide did not show a characteristic absorption peak, then it did not contain protein ingredients.

#### 2.4.2. Ninhydrin Method

A drop of 1.0 mg/mL polysaccharide solution was dropped onto a filter paper and dried. Then, 2.5% ninhydrin was sprayed on the paper. If the paper maintained its original pale-yellow color, then the sample did not contain proteins or amino acids; if the paper became purple, the sample contained proteins or amino acids [17].

### 2.5. Analysis of Sulfate-Group Content

The sulfate-group (–SO_3_H) content of polysaccharides was determined by the BaCl_2_-gelatin turbidity method [18]. In a typical procedure, 0.3% gelatin solution was prepared in hot water (60–70 °C) and stored at 4 °C overnight. Two grams of BaCl_2_ was dissolved in gelatin solution and allowed to stand for 2–3 h at 25 °C. About 0.20 mL of polysaccharide solution (1.4 mg/mL) was added to 3.8 mL of 0.5 M HCl and 1 mL of BaCl_2_-gelatin reagent, and the mixture was allowed to stand for 10–20 min. A blank was prepared with 0.2 mL of water instead of SPS solution. The released barium sulfate suspension was measured at λ = 360 nm by UV–VIS spectrophotometry using potassium sulfate as standard, and the standard curve was shown in Figure S1. This experiment was performed in triplicate.

### 2.6. Analysis of Carboxylic Group Content

**Figure 1:**
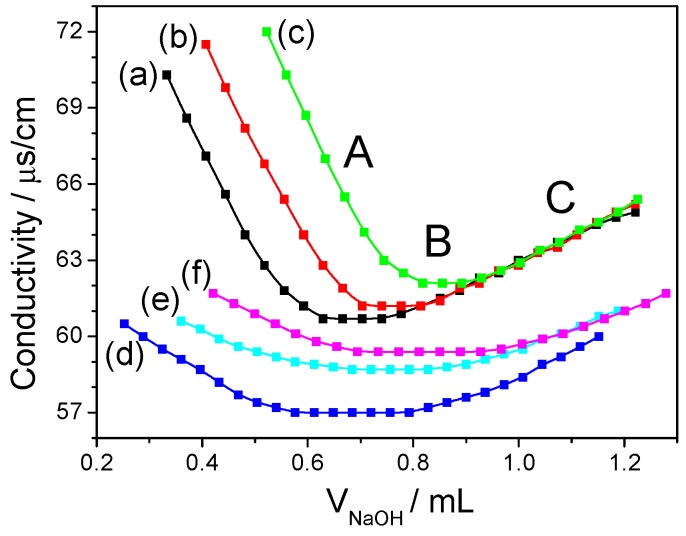
Conductivity curve of –COOH content of *Porphyra*-2 polysaccharide before and after degradation. (**A**) electrical conductivity decreasing stage; (**B**) equilibrium stage; (**C**) electrical conductivity increasing stage. (**a**–**c**) are the triplicate experiments before degradation; (**d**–**f**) are three parallel experiments after degradation.

The carboxylic group (–COOH) content of SPS was measured by conductometric titration [19]. A conductivity titration curve was drawn using the value of conductivity as the *Y*-axis and the corresponding volume of the used NaOH as the *X*-axis. The conductivity titration curve could be divided into three sections (Figure 1): the conductivity decreasing stage (A), the equilibrium stage (B), and the conductivity increasing stage (C). Three tangent lines were constructed from the curve of three stages, and the cross-points were the stoichiometric points. The cross-point of lines A and B gave the volume of NaOH (*V*_1_) that excess hydrochloric acid and –SO_3_H consumed; and the cross-point of lines B and C gave the volume of NaOH (*V*_2_) that excess hydrochloric acid and the –COOH of the polysaccharides consumed together, so *V*_2_ – *V*_1_ (platform portion) was the volume of NaOH that the –COOH of SPSs consumed alone. The –COOH content could be obtained according to the following formula:

(1) $$–\mathrm{COOH}\;(\%)=\frac{C_{NaOH}\times (V_{2}-V_{1})\times 45/1000}{C_{\text{sample}}\times 40/1000}\times 100$$

where *C*_NaOH_ (mol/L) represents the molar concentration of NaOH, *C*_Sample_ (g/L) represents the mass concentration of polysaccharide, 45 is the molar mass of –COOH and 40 (mL) is the volume of polysaccharide solution. The final value was the average of three parallel experiments.

### 2.7. Fourier Transform Infrared Spectrometry

A dried polysaccharide sample (2.0 mg) was mixed with KBr (200 mg). After grinding and pressing into a KBr pellet, scanning was performed between the ranges of 4000–400 cm^−1^ wave number.

### 2.8. Cytotoxicity Measurement of Polysaccharides

HK-2 cells were cultured in a DMEM culture medium containing 10% fetal bovine serum, 100 U/mL penicillin-100 μg/mL streptomycin antibiotics with pH 7.4 at 37 °C in a 5% CO_2_ humidified environment. Upon reaching 80%–90% confluent, cells were blown gently after trypsin digestion to form cell suspension for the following cell experiment.

To assess the cytotoxicity of polysaccharides as other authors have already performed [20,21,22], the cytotoxicities of six samples were determined as follows: Cell suspension with a cell concentration of 1 × 10^5^ cells/mL was inoculated per well in 96-well plates for 24 h. After the cells were confluent, the culture medium was removed by suction and the cells were washed twice with PBS. After that, 0.1 mL of 0, 0.1, 1, 10, and 100 μg/mL of different seaweed polysaccharides solutions were added respectively. Each experiment was repeated in three parallel wells. After incubation for 24 h, 10 μL CCK-8 was added to each well and incubated for 1.5 h. The OD values were measured using the enzyme mark instrument at 450 nm. Cell viability was determined using the following equation: Cell viability (%) = OD_treatment group_/OD_control group_ × 100.

### 2.9. Repair Effect of Polysaccharide on Damaged HK-2 Cells

Cell suspension with a cell concentration of 1 × 10^5^ cells/mL was inoculated per well in 96-well plates, 0.1 mL of DMEM containing 10% fetal bovine serum was added per well and the cells were incubated for 24 h. The experimental model was divided into three groups [23]: (1) Control group: in which only serum-free culture medium was added; (2) Damaged group: in which serum-free culture medium with 2.8 mmol/L oxalate was added and incubated for 3 h; (3) Repair group: in which 60 μg/mL of different SPS solutions were added into the cells of damaged control group and repaired for 8 h. After completing the repairing, 10 μL of CCK-8 was added to each well and incubated for 1.5 h. The OD values were measured using the enzyme mark instrument at 450 nm to detect the repair capacity of polysaccharide.

### 2.10. Hematoxyline-Eosin Staining

HK-2 cells were grouped as in section 2.9. After the repair was completed, the supernatant was removed by suction and washed 2–3 times with PBS. Afterwards, the cells were fixed with 3.8% polyoxymethylene for 15 min at room temperature. Cells were washed three times with PBS. After fixation the cells were stained with hematoxylin stain and incubated for 15 min. Then cells were washed with distilled water (DW) for 2 min to remove excess stain. After that, the cells were stained with eosin staining solution for 5 min. The cells were washed with DW for 2 min to remove excess eosin. After treatment, the cells were observed under the microscope, the nucleus of the cells was stained purple or blue, whereas the cytoplasm was stained pink or red.

### 2.11. Statistical Analysis

Results were expressed as Mean ± SD from three independent experiments. The statistically significant differences were analyzed using Turkey’s test. *p* values < 0.05 were considered statistically significant, and *p* < 0.01 considered extremely significant.

## 3. Results and Discussion

### 3.1. Degradation of Polysaccharides

Sulfated polysaccharides from different seaweeds were selected for this study and further degraded using H_2_O_2_. The natural polysaccharides were degraded for two reasons. First, the high molecular mass of natural polysaccharides hindered the biological activity exerted by polysaccharides. This is supported by a number of previous studies where activities of low-molecular-weight polysaccharides after degradation were often better than before [24,25]. Second, in the present work, we aimed to compare the ability of polysaccharides with different –SO_3_H contents to repair damaged renal epithelial cells, and the molecular weight of polysaccharides must be controlled to within a narrow range, thereby ensuring the accuracy of experimental research. This is confirmed by Zhao *et al.* [10], who suggested that when the molecular weight and molecular structure of polysaccharides were similar, the sulfate content of polysaccharides was the dominant factor affecting their biochemical activities. Therefore, polysaccharide degradation was necessary.

Accordingly, the initial molecular weight of some polysaccharide molecules was very high, the natural polysaccharides were degraded under different conditions (Table 1) to give the molecular weight of all polysaccharides with a narrow differences (from 1968 to 4020 Da). The molecular weight of polysaccharides was influenced by various degradation factors, such as H_2_O_2_ concentration, reaction temperature, and time. By changing H_2_O_2_ concentration in the degradation step, a suitable molecular weight of the polysaccharide can be obtained.

### 3.2. Chemical Characteristics

The molecular weights of six natural polysaccharides and four degraded polysaccharides are shown in Table 2. Although the molecular weights of all polysaccharides were significantly different before degradation, they had narrow differences (from 1968 to 4020 Da) after degradation under different conditions. The viscosity of all degraded polysaccharides decreased significantly (Table 1) and solubility increased compared with undegraded polysaccharides.

The –SO_3_H content of six kinds of natural SPSs ranged between 6.1%–21.7% (Table 2). Laminarin-1 contained the highest (21.7%) –SO_3_H content, whereas *Undaria*-6 (6.1%) contained the lowest. The –SO_3_H contents of polysaccharides were not significantly changed after degradation, which indicated that degradation did not exert a considerable impact on the –SO_3_H contents of polysaccharides.

The –SO_3_H contents of degraded *Porphyra*-2 and *Gracilaria*-3 (17.9% and 13.3%, respectively) were slightly higher than that of undegraded samples (17.3% and 12.7%, respectively). The hydroxyl radicals generated by the H_2_O_2_ system broke the sugar chains to expose the –SO_3_H inside the polysaccharide. This phenomenon led to higher levels of –SO_3_H content detected in the low-molecular-weight fragments after degradation [26]. Conversely, the –SO_3_H content (5.5%) of degraded *Undaria*-6 was found to be slightly lower than that of undegraded fragment (6.1%) because desulfonation frequently occurs in acidic medium or at elevated temperature [4]. In the present work, degradation was carried out in a weak acid medium of H_2_O_2_ at 90 °C, which may have led to the slight decrease in –SO_3_H content of SPSs.

The –COOH contents of all natural and degraded polysaccharides were measured by conductometric titration. The conductivity curves are illustrated in Figure 1, and the obtained value of uronic acid and conversion of uronic acid content are shown in Table 2. Akahane *et al.* [27] measured sulfate- and carboxyl-group content of a mixture alginic acid (–COOH only) and dextran sulfate (–SO_3_H only) with different proportions using the conductometric titration method. They found that both groups do not interfere with each other. Luo *et al.* [19] calculated the sulfate- and carboxyl- group content of sea-cucumber polysaccharide by conductometric titration. They showed that conductometric titration can simultaneously determine –SO_3_H and –COOH contents, but the curves of both groups differ in inflexion point. However, in the current study, we determined only –COOH content through the conductometric titration method. The –COOH content of six natural SPSs varied between 0.9% and 1.4%, in which *Sargassum*-4 (1.4%) had the highest –COOH content, and *Porphyra*-2 (0.9%) had the lowest. The –COOH content of SPSs only slightly changed after degradation.

All natural and degraded polysaccharides did not show any characteristic absorption peak at 260–280 nm and did not demonstrate a purple color with ninhydrin. Thus, all of them were devoid of proteins.

### 3.3. FT-IR Spectroscopy

**Figure 2:**
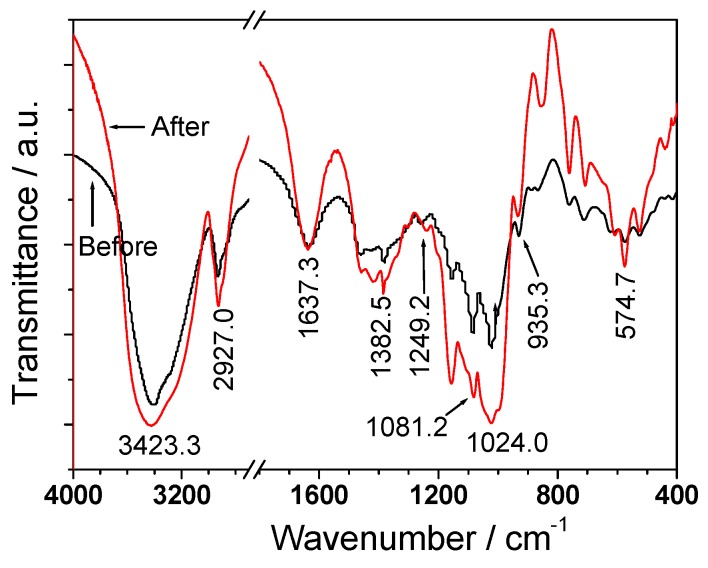
FT-IR spectra of representative seaweed polysaccharide degraded *Sargassum*-4.

Figure 2 shows the FT-IR spectrum of one representative algal polysaccharide. The FT-IR spectra of other polysaccharides are showed in Figure S2. The FT-IR spectra of all other SPSs were similar, and the main absorption peaks of the degraded and undegraded SPSs are shown in Table 3. A wide peak at about 3399.9–3429.2 cm^−1^ was due to the hydroxyl stretching vibration, and the peak at 2912.2–2939.6 cm^−1^ was due to C–H stretching vibration. The peak of 1380.2–1384.9 cm^−1^ could be assigned to deforming vibrations of the C–H bond [28]. The peak at about 1614.7–1637.3 cm^−1^ was attributed to the asymmetric and symmetric stretching vibrations of COOH. The peak at 1459.1 cm^−1^ was due to C–OH deformation vibration with contribution of O–C–O symmetric stretching vibration of carboxylate group. The peak at 1249.2–1262.8 cm^−1^ was due to the stretching vibration of S=O [29]. The peaks at about 1080.9–1086.9 and 1019.4–1025.6 cm^−1^ could be assigned to the stretching vibration of C–O. The peak at 931.8–935.3 cm^−1^ was attributed to the stretching vibration of C=O in uronic acid [30]. The peak at 881.2 cm^−1^ could be assigned to the C–H scissor vibration of β-type heterogeneous glycosidic bond [31].

The FT-IR spectrum of the degraded polysaccharide was very close to the undegraded one, which indicated that the overall structure of polysaccharide was not significantly affected by H_2_O_2_ degradation. However, the oxidative degradation of polysaccharides by H_2_O_2_ may result in the scission of carbohydrate chains and depolymerized polysaccharides by breaking glycosidic linkages [32]. Moreover, the –SO_3_H peak intensities of the four kinds of degradation products did not change after degradation, indicating that the –SO_3_H contents of all degraded polysaccharides had no significant variation. The relationship between the content of –SO_3_H in seaweed polysaccharides and the intensity of –SO_3_H absorption peak in FT-IR is shown in Figure S3 in the Supplementary Materials.

### 3.4. Cytotoxicity of Different SPSs on HK-2 Cells

**Figure 3:**
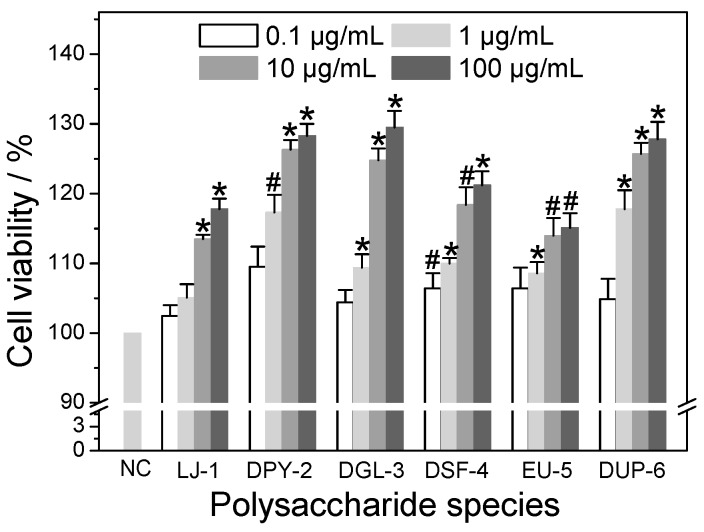
Cytotoxic effect of six low-molecular-weight polysaccharides with different contents of –SO_3_H group on HK-2 cells. Cells were incubated with different concentration of (0.1–100 μg/mL) polysaccharides for 24 h and then cell viability was determined by CCK-8 assay. The control represents 100% cell viability. Data are reported as Mean ± SD (*n* = 3), derived from three independent experiments. Compared with normal control group, ^#^ indicates *p* < 0.05, * indicates *p* < 0.01. NC: Normal control; LJ-1: *Laminaria*-1; DPY-2: degraded *Porphyra*-2; DGL-3: degraded *Gracilaria*-3; DSF-4: degraded *Sargassum*-4; EU-5: *Eucheuma*-5; DUP-6: degraded *Undaria*-6.

The cytotoxicities of six kinds of SPSs with different contents of –SO_3_H group on normal HK-2 cells were detected by the CCK-8 method (Figure 3). When HK-2 cells were exposed to different concentrations of polysaccharides (0.1, 1, 10, and 100 μg/mL) for 24 h, the cell viability of all samples was above 100% and increased with increased polysaccharide concentration, indicating that these polysaccharides did not express any cytotoxicity within the range 0.1–100 μg/mL and also promoted cell growth. Possibly, the SPSs provided nutrition to cells, as cells divided rapidly due to sufficient nutrition and space. Therefore, cell viability was increased at the end of the assay. Similarly, Liang *et al.* [33] revealed that red SPSs (ι-carrageenan) exhibited no cytotoxicity and promoted the proliferation of human umbilical vein endothelial cells (HUVEC) within the range 5–1000 μg/mL, and the cell viability was more than 140% at the concentration of 800 μg/mL. Dore *et al.* [34] also found that the *Sargassum vulgare* polysaccharide increased the viability of normal rabbit aortic endothelial cells to above 100% in the concentration range of 25–100 μg/mL, and also promoted cell growth. Yao *et al.* [35] reported that *Aloe* polysaccharide speeded up the proliferation of fibroblasts significantly within the concentration range of 25–400 μg/mL.

### 3.5. Repair Ability of Different SPSs on Damaged HK-2 Cells

#### 3.5.1. Effect of Polysaccharide Concentration

**Figure 4:**
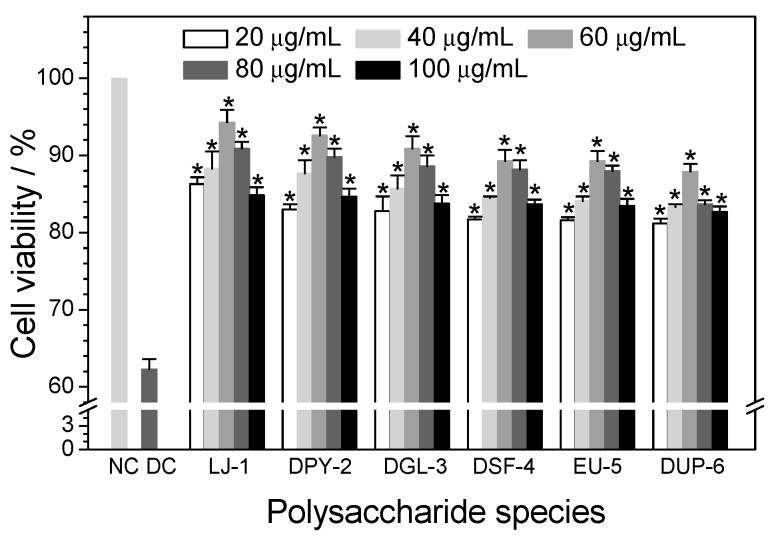
Effect of different polysaccharide concentrations (20, 40, 60, 80, and 100 μg/mL) on the viability of oxalate-induced damaged HK-2 cells. The cells were incubated with 2.8 mmol/L oxalate for 3 h, and then treated with different concentrations of SPSs for 8 h. Afterwards, cell-viability was determined by CCK-8 assay. Each bar was derived from three independent experiments and data are reported as Mean ± SD. Compared with damaged control group, * indicates *p* < 0.01. NC: Normal control; DC: Damaged control; LJ-1: *Laminaria*-1; DPY-2: degraded *Porphyra*-2; DGL-3: degraded *Gracilaria*-3; DSF-4: degraded *Sargassum*-4; EU-5: *Eucheuma*-5; DUP-6: degraded *Undaria*-6.

To optimize the repair effect of six SPSs on damaged HK-2 cells induced by 2.8 mmol/L oxalate, we performed a concentration-depended experiment in the concentration range from 20 to 100 μg/mL for 8 h (Figure 4). Oxalate is toxic to renal tubular cells by inducing inflammation, membrane damage, oxidative cell stress, DNA synthesis, and necrosis cell death at high concentrations (≥5 mmol/L) [36]. Figure 4 shows that all polysaccharides considerably repaired damaged cells compared with the damaged control group. Simultaneously, when damaged cells were repaired by different concentrations (20, 40, 60, 80, and 100 μg/mL) of polysaccharides, the cell viability of damaged cells was initially increased, reaching the maximum at 60 μg/mL, and then decreasing at higher concentrations (100 μg/mL), indicating that 60 μg/mL was adequate for the polysaccharides to play a role. Moreover, it was a remarkable fact that, although results of cell-viability between 60 and 80 μg/mL were similar for both degraded *Sargassum*-4 and *Eucheuma*-5, greater differences were found between results of 60 and 80 μg/mL for *Laminaria*-1 and degraded *Undaria*-6. Thus, in subsequent experiments, 60 μg/mL polysaccharides was chosen to repair cells and establish the effect of six SPSs to repair oxalate-induced damaged cells.

#### 3.5.2. Effect of –SO_3_H Content

**Figure 5:**
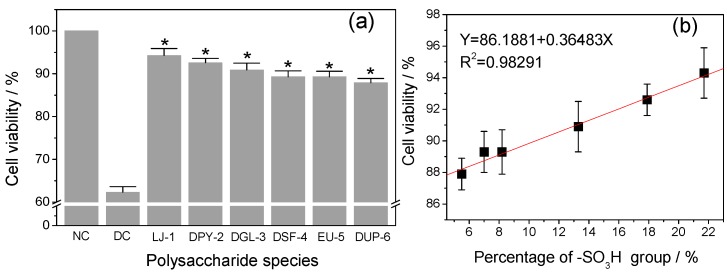
Repair capacity of six low-molecular-weight seaweed polysaccharides (SPSs) on oxalate-induced damaged HK-2 cells (**a**), and the relationship between –SO_3_H content and repair capacity of SPSs (**b**). Each bar is derived from three independent experiments and data are reported as Mean ± SD. Compared with damaged control group, * indicates *p* < 0.01. NC: Normal control; DC: Damaged control; LJ-1: *Laminaria*-1; DPY-2: degraded *Porphyra*-2; DGL-3: degraded *Gracilaria*-3; DSF-4: degraded *Sargassum*-4; EU-5: *Eucheuma*-5; DUP-6: degraded *Undaria*-6.

Six low-molecular-weight polysaccharides with narrow differences but different –SO_3_H contents were used to repair oxalate-induced damaged HK-2 cells. Results are shown in Figure 5a. All six polysaccharides at 60 μg/mL exerted a repair effect on injured cells to various degrees because the polysaccharides contained the negatively charged –SO_3_^−^ and –COO^−^ groups, which could reduce the loss of the negative charge on the cell surface and repair the charge barrier [37]. Sun *et al.* [26] confirmed that the inhibitory effect of polysaccharides from two marine *Chrysophyta* on the hemolysis of RBCs may be due to the reducibility groups such as hydroxyl, sulfate, and uronic acid groups of the polysaccharides. These negatively-charged groups combine with the free radicals and restore the activity of cells. SPSs are exogenous antioxidants enhancing the antioxidant activity of cells by reducing free-radical generation and oxidative stress in renal epithelia cells. Hence, SPSs can protect and repair renal cells by increasing antioxidant capacity and blocking the injury pathway, which prevents the binding and retention of CaO*x* crystals to renal tissue, eventually reducing the occurrence of kidney-stone diseases. In the present work, when injured cells were repaired by the polysaccharides *Laminaria*-1, degraded *Porphyra*-2, degraded *Gracilaria*-3, degraded *Sargassum*-4, *Eucheuma*-5, and degraded *Undaria*-6 with different –SO_3_H contents (21.7%, 17.9%, 13.3%, 8.2%, 7.0%, and 5.5%, respectively), the cell viabilities of injured cells increased from 62.3% (damaged control) to 94.3%, 92.6%, 90.9%, 89.3%, 89.3%, and 87.9%, respectively. Meanwhile, results suggested that samples with higher –SO_3_H content had the maximum capacity to repair damaged cells, whereas samples with lower –SO_3_H content had modest activity. However, when compared the repair ability of all polysaccharides among each other, we did not find significant difference at *p* < 0.05. Furthermore, as aforementioned, the activity of polysaccharides also depended on –COOH content. Given that our samples had similar –COOH contents (between 1.0% and 1.7%), the effect of –COOH content could be avoided. Therefore, the repair abilities of polysaccharides were confirmed to be closely related to –SO_3_H content (Figure 5b). This finding was supported by the findings of Song *et al.* [38] on three polysaccharide fractions (B1, B2, and B3) from *Bryopsis plumose* with different –SO_3_H contents (7.56%, 6.71%, and 11.4%, respectively) that B3 with the highest –SO_3_H group content has the highest IC_50_ values 9.2 and 1.7 mg/mL in the scavenging activity of superoxide anion and DPPH free radicals, respectively. Therefore, the antioxidant ability of the three components was proportional to –SO_3_H content.

#### 3.5.3. Effect of Molecular Weight

**Figure 6:**
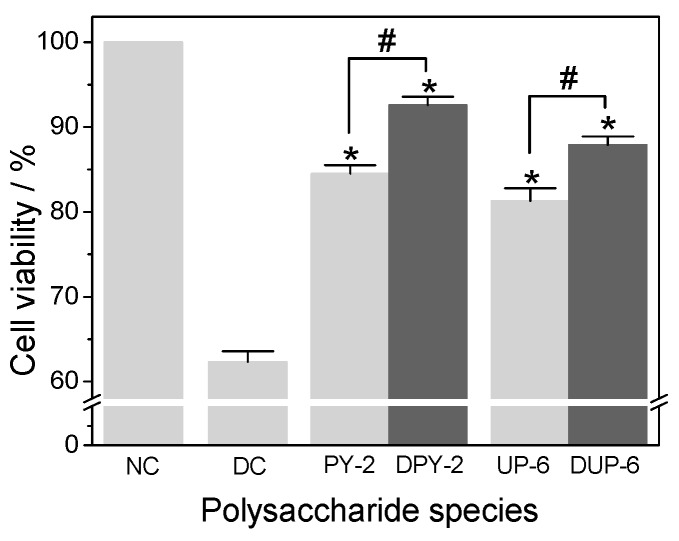
Cell repair activity of selected polysaccharides of different molecular weight on damaged HK-2 cells. Data were expressed as Mean ± SD from three independent experiments. Compared with damaged control group, * indicates *p* < 0.01. ^#^ indicates *p* < 0.05. NC: Normal control; DC: Damaged control; LJ-1: *Laminaria*-1; DPY-2: degraded *Porphyra*-2; DGL-3: degraded *Gracilaria*-3; DSF-4: degraded *Sargassum*-4; EU-5: *Eucheuma*-5; DUP-6: degraded *Undaria*-6.

The influence of molecular weight of polysaccharides on the repair of oxalate-induced damaged cells is shown in Figure 6. Based on the –SO_3_H content, *Porphyra*-2 and *Undaria*-6 were selected to test the effect of molecular weight, because *Porphyra*-2 contained the highest –SO_3_H content, whereas *Undaria*-6 contained the lowest among the degraded samples. After depolymerization, the molecular weight of the polysaccharides *Porphyra*-2 and *Undaria*-6 decreased from 3.1 × 10^4^ to 4020 Da and 5.2 × 10^6^ to 3635 Da, respectively. However, both samples had similar –SO_3_H and –COOH contents before and after degradation (Table 2). Figure 6 suggests that the lower-molecular-weight fragments of *Porphyra*-2 and *Undaria*-6 exhibited stronger damage repair activity (92.6% and 87.9%, respectively) in HK-2 cells than their native polysaccharides (84.5% and 81.3%, respectively). There was significant difference occurred between *Porphyra*-2 before and after degradation (*p* < 0.05), and also for *Undaria*-6 before and after degradation (*p* < 0.05). In other words, degraded SPSs with low molecular weights showed better repair abilities. This finding corresponded with that of a previous study where low-molecular-weight fragments of *Porphyridium cruentum* polysaccharides after degradation showed stronger antioxidant activity than high-molecular-weight samples [39]. Clearly, the repair activity of polysaccharides was inversely related to their molecular weight. It was supposed that low molecular weight polysaccharides have more reductive hydroxyl group terminals to accept and remove the free radicals generated by oxalate [40]. However, it was not conducive for high molecular weight polysaccharides to cross cell-membranes, so it was difficult to exert their biological activities.

### 3.6. Morphological Changes of HK-2 Cells after Repair by Different Polysaccharides

**Figure 7:**
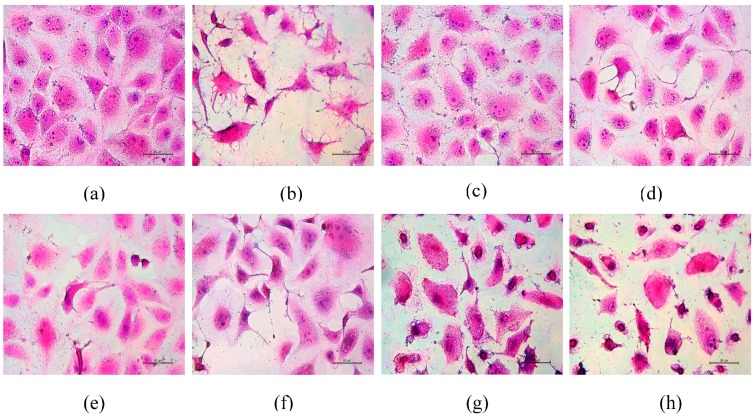
Repair effect of six SPSs on the morphology of damaged HK-2 cells evaluated by hematoxylin and eosin (HE) staining: (**a**) normal control; (**b**) damaged HK-2 cells; (**c**) *Laminaria*-1; (**d**) degraded *Porphyra*-2; (**e**) degraded *Gracilaria*-3; (**f**) degraded *Sargassum*-4; (**g**) *Eucheuma*-5; and (**h**) degraded *Undaria*-6. Oxalate-induced damaged HK-2 cells were treated with 60 μg/mL polysaccharides for 8 h, and then cell morphology was determined by HE staining under a microscope. The nucleus was stained blue or purple, and the cytoplasm was pink or red. Magnification: 400×.

The repair effect of the six SPSs on the morphology of damaged HK-2 cells was evaluated by hematoxylin and eosin staining. As shown in Figure 7a, HK-2 cells of the normal control group exhibited normal morphologies with an oval shape and round nucleus. Moreover, the integrity of tight junctions between cells and microvilli were clearly visible. When HK-2 cells were exposed to oxalate (2.8 mmol/L) for 3 h, the cells lost their natural shape and were shrunken into fibroblast like a spindle, cell connections were broken, and nuclear condensation and apoptotic bodies were clearly observed with a dense stain (Figure 7b). These abnormal morphological findings suggested that oxalate damaged the renal membrane, which may contribute to the development of stone disease by altering the properties of the renal epithelial cell membrane. After treating the damaged cells with various algal polysaccharides, cell morphology was gradually restored, and cellular connections tightened (Figure 7c–f), whereas *Eucheuma*-5 and degraded *Undaria*-6 showed less effect (Figure 7g,h) compare to other SPSs because of their low sulfate contents. All evaluated polysaccharide samples were able to restore the structure of damaged cells to varying degrees compared with the damaged control group. *Laminaria*-1 polysaccharide showed increased repair effect on cell morphology compared with all other polysaccharides. The cell-morphology-restoration activity of the other five components decreased in the order degraded *Porphyra*-2 > degraded *Gracilaria*-3 > degraded *Sargassum*-4 > *Eucheuma*-5 > degraded *Undaria*-6, the same as the order of sulfate contents of the samples. This finding indicated that –SO_3_H content affected the repair ability of polysaccharides, in accordance with the result of cell-viability assay.

## 4. Conclusions

Six types of low-molecular-weight seaweed polysaccharides with narrow differences (from 1968 to 4020 Da) but different contents of the characteristic –SO_3_H group (21.7%, 17.9%, 13.3%, 8.2%, 7%, and 5.5%, respectively) were selected to examine their impact on oxalate-induced damaged HK-2 cells. These polysaccharides did not express cytotoxicity within the 0.1–100 μg/mL dose range. When they were used to repair the damaged HK-2 cells induced by oxalate, the repair abilities were positively associated with the –SO_3_H content of polysaccharide and negatively associated with molecular weight. The polysaccharides had the best repair effect at 60 μg/mL. Thus, seaweed sulfated polysaccharides can be used to prevent or treat hyperoxaluria and renal-stone formation.

## Figures and Tables

**Table 1:** Degradation conditions and physico-chemical properties of different seaweed polysaccharides (SPSs).

Polysaccharide species	Concentration of H_2_O_2_	Constant of Mark-Houwink equation		Intrinsic viscosity η (mL/g)	
		κ	α	Before degradation	After degradation
LJ-1	–	8.21 × 10^−3^	0.782	3.1	–
PY-2	12%	7.86 × 10^−3^	0.626	5.1	1.4
GL-3	10%	7.00 × 10^−2^	0.720	1,039	24.1
SF-4	12%	1.92 × 10^−4^	1.23	26.3	4.9
EU-5	–	1.92 × 10^−4^	1.23	3.4	–
UP-6	15%	8.21 × 10^−3^	0.782	1460	4.9

LJ-1: *Laninaria*-1, PY-2: *Porphyra*-2, GL-3: *Gracilaria*-3, SF-4: *Sargassum*-4, EU-5: *Eucheuma*-5, UP-6: *Undaria*-6.

**Table 2:** Molecular weight, –SO_3_H content, and –COOH content of native and degraded SPSs.

SPSs	Mean molecular weights *M*/Da		–SO_3_H contents/%		–COOH contents/%		Uronic acid contents/%	
	BD *	AD *	BD	AD	BD	AD	BD	AD
*Laminaria*-1	1,968	–	21.7	–	1.2	–	5.2	–
*Porphyra*-2	3.1 × 10^4^	4,020	17.3	17.9	0.9	1.7	3.9	7.3
*Gracilaria*-3	6.2 × 10^5^	3,343	12.7	13.3	1.0	1.0	4.3	4.3
*Sargassum*-4	1.6 × 10^4^	3,828	8.4	8.2	1.4	1.3	6.0	5.4
*Eucheuma*-5	2,850	–	7.0	–	1.2	–	5.0	–
*Undaria*-6	5.2 × 10^6^	3,635	6.1	5.5	1.3	1.2	5.6	5.2

BD *: before degradation; AD *: after degradation.

**Table 3:** FT-IR characteristic absorption peaks of native and degraded seaweed polysaccharides.

SPSs	–SO_3_H contents/%	–COOH contents/%	Characteristic absorption peaks of groups/cm^−1^			
			–OH	–COOH	–OSO_3_	Sugar ring
LJ-1	21.7	1.2	3,400.9	1,632.1	1,262.3	2,912.2, 1,460, 1,380.3, 1,082.5, 1,019.4, 881.2
PY-2	17.3	0.9	3,436.3	1,632.9	1,249.2	2,922.0, 1,383.3, 1,080.9
DPY-2	17.9	1.7	3,429.2	1,618.4	1,250.0	2,925.5, 1,384.9, 1,080.8
GL-3	12.7	1.0	3,419.2	1,641.1	1,257.2	2,921.5, 1,378.4, 1,024.3
DGL-3	13.3	1.0	3,415.3	1,614.7	1,261.1	2,921.4, 1,383.8, 1,025.6
SF-4	8.4	1.4	3,415.1	1,630.9	1,260.2	2,931.8, 1,460.0, 1,380.2, 1,084.4, 1,021.6, 928.1
DSF-4	8.2	1.3	3,423.3	1,637.3	1,247.7	2,927.0, 1,421.2, 1,382.5, 1,081.2, 1,024.0, 935.3
EU-5	7.0	1.2	3,399.9	1,629.5	1,256.3	2,939.6, 1,459.1, 1,381.1, 1,086.9, 1,022.0, 931.8
UP-6	6.1	1.3	3,419.4	1,632.7	1,262.3	2,917.3, 1,022.5
DUP-6	5.5	1.2	3,413.2	1,615.8	1,262.8	2,921.4, 1,024.7

LJ-1: *Laninaria*-1; PY-2: *Porphyra*-2; DPY-2: Degraded *Porphyra*-2; GL-3: *Gracilaria*-3; DGL-3: Degraded *Gracilaria*-3; SF-4: *Sargassum*-4; DSF-4: Degraded *Sargassum*-4; EU-5: *Eucheuma*-5; UP-6: *Undaria*-6; DUP-6: Degraded *Undaria*-6.

## Supplementary Materials

Supplementary Materials can be found at www.mdpi.com/2073-4360/8/5/188/s1.
