# Supplementary material for: Repair Effect of Seaweed Polysaccharides with Different Contents of Sulfate Group and Molecular Weights on Damaged HK-2 Cells
Source: Polymers (Basel). 2016 May 19;8(5):188. doi: 10.3390/polym8050188 (PMC6431945; doi:10.3390/polym8050188)
Supplement: Supplementary file 1 [file polymers-08-00188-s001.pdf]

Supplementary Materials: Repair Effect of Seaweed Polysaccharides with Different Contents of Sulfate Group and Molecular Weights on Damaged HK-2 Cells

Poonam Bhadja, Cai-Yan Tan, Jian-Ming Ouyang and Kai Yu

1. Standard Curve for Analysis of Sulfate Group Content

The sulfate content of polysaccharides was determined by the $BaCl_2$-gelatin turbidity method [18]. In a typical procedure, 0.3% gelatin solution was prepared in hot water (60–70 °C) and stored at 4 °C overnight. Two grams of $BaCl_2$ was dissolved in gelatin solution and allowed to stand for 2–3 h at 25 °C. About 0.20 mL of polysaccharide solution (1.4 mg/mL) was added to 3.8 mL of 0.5 M HCl and 1 ml of $BaCl_2$-gelatin reagent, and the mixture was allowed to stand for 10–20 min. A blank was prepared with 0.2 ml of water instead of SPS solution. The released barium sulfate suspension was measured at $\lambda = 360$ nm by UV–VIS spectrophotometry using potassium sulfate as standard.

The standard curve was shown in Figure S1. The obtained regression equation is: $Y = 0.01042 + 1.27905X$, $n = 11$, $R = 0.99324$ (Figure 1).

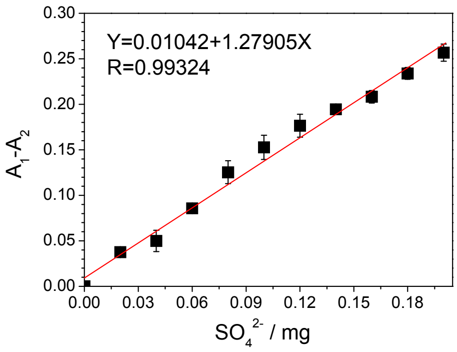

Figure S1. Standard curve for determination of sulfate group content in seaweed polysaccharides by $BaCl_2$- gelatin method. Regression equation is: $Y = 0.01042 + 1.27905X$, $R = 0.99324$.

2. FT-IR Spectra of Six Low-Molecular-Weight Polysaccharides

Figure S2 shows the FT-IR spectra of the six algal polysaccharides. FT-IR spectra suggested a positive correlation between the absorption peak intensity of $–SO_3H$ (1249.2–1262.8 cm^{-1}) and $–SO_3H$ content of polysaccharides (Figure S3). For instance, peak intensity of $–SO_3H$ of Laminarin-1 was the strongest, and its $–SO_3H$ content was the highest (21.7%). An approximate linear relationship between the peak intensities of $–SO_3H$ and $–SO_3H$ contents can be obtained (Figure S3).

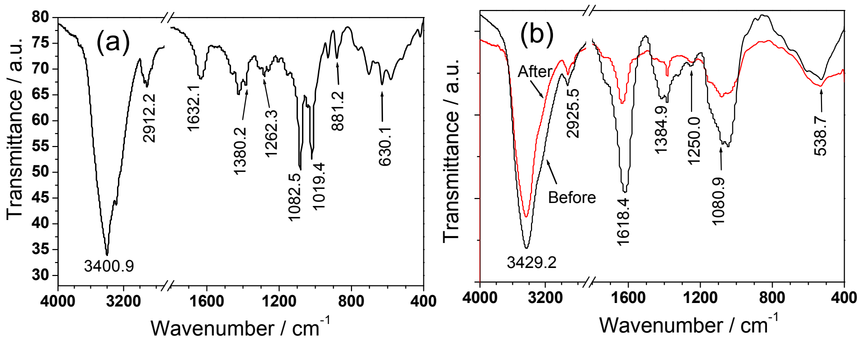

Figure S2. *Cont.*

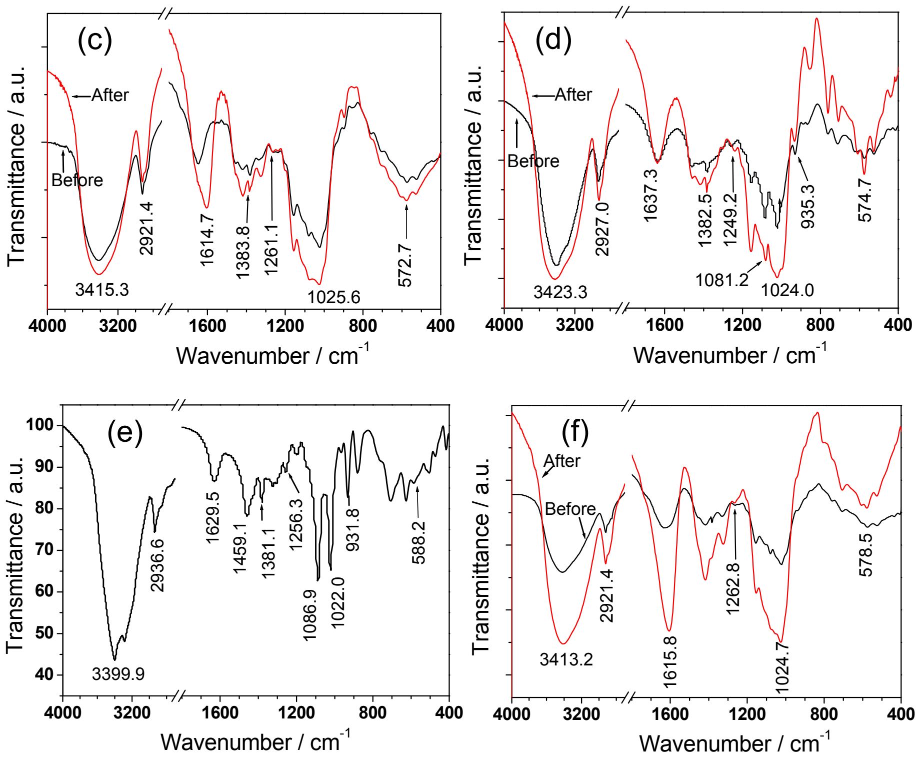

Figure S2. FT-IR spectra of (**a**) *Laminaria*-1; (**b**) degraded *Porphyra*-2; (**c**) degraded *Gracilaria*-3; (**d**) degraded *Sargassum*-4; (**e**) *Eucheuma*-5; and (**f**) degraded *Undaria*-6 polysaccharides.

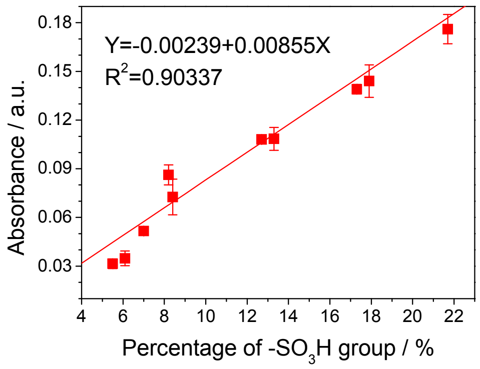

Figure S3. Relationship between the content of $–SO_3H$ in seaweed polysaccharides and the intensity of $–SO_3H$ absorption peak in FT-IR.

© 2016 by the authors; licensee MDPI, Basel, Switzerland. This article is an open access article distributed under the terms and conditions of the Creative Commons Attribution (CC-BY) license (http://creativecommons.org/licenses/by/4.0/).
